# Regulation of mitochondrial trafficking, function and quality control by the mitochondrial GTPases Miro1 and Miro2

**DOI:** 10.1186/2193-1801-4-S1-L33

**Published:** 2015-06-12

**Authors:** Josef Kittler

**Affiliations:** University College London, UK

**Keywords:** Mitochondria, mitophagy, parkinson’s

Regulated trafficking of mitochondria in neurons is essential for providing ATP at the correct spatial location to power neural function and computation, and for providing calcium buffering at sites of calcium entry or release. Indeed the regulation of mitochondrial distribution, morphology and function are proposed to play an important role in neuronal development and survival but the regulatory mechanisms remain unclear. Miro family proteins (Miro1 and Miro2 in mammals) contain a transmembrane domain locating them to the outer mitochondrial membrane, along with two GTPase domains and two calcium-sensing EF-hand domains that face into the cytosol, and play a key role in regulating mitochondrial transport. Miro proteins mediate mitochondrial trafficking in neurons by linking mitochondria to kinesin and dynein motor proteins for their transport in axons and dendrites. Miro proteins are also targets for the Parkinson’s Disease associated PINK1/Parkin mitophagy pathway and are therefore implicated in altered mitochondrial dynamics during mitophagy. Here I will present our recent results on the role played by Miro proteins in regulating mitochondrial trafficking and quality control. The role that Miro-mediated control of mitochondrial trafficking and turnover plays in regulating neuronal development, function and pathology will also be explored.

